# IL-12 Can Target Human Lung Adenocarcinoma Cells and Normal Bronchial Epithelial Cells Surrounding Tumor Lesions

**DOI:** 10.1371/journal.pone.0006119

**Published:** 2009-07-01

**Authors:** Irma Airoldi, Emma Di Carlo, Claudia Cocco, Emanuela Caci, Michele Cilli, Carlo Sorrentino, Gabriella Sozzi, Silvano Ferrini, Sandra Rosini, Giulia Bertolini, Mauro Truini, Francesco Grossi, Luis Juan Vicente Galietta, Domenico Ribatti, Vito Pistoia

**Affiliations:** 1 A.I.R.C. Tumor Immunology Unit, Department of Experimental and Laboratory Medicine, G. Gaslini Institute, Genova, Italy; 2 Department of Oncology and Neurosciences, “G. d'Annunzio” University and Ce.S.I. Aging Research Center, “G. d'Annunzio” University Foundation, Chieti, Italy; 3 Laboratory of Molecular Genetics, Istituto Giannina Gaslini, Genova, Italy; 4 Animal Model Facility, Istituto Nazionale per la Ricerca sul Cancro, Genova, Italy; 5 Cytogenetic and Molecular Cytogenetic Department, Fondazione IRCCS Istituto Nazionale Tumori, Milan, Italy; 6 Department of Translational Oncology, Istituto Nazionale per la Ricerca sul Cancro, Genova, Italy; 7 Anatomic Pathology Section, “SS Annunziata” Hospital, Chieti and Ce.S.I. Aging Research Centre, “G. d'Annunzio” University Foundation, Chieti, Italy; 8 Department of Pathology, Istituto Nazionale per la Ricerca sul Cancro, Genova, Italy; 9 Oncologia Medica A, Disease Management Team, Lung Cancer, Istituto Nazionale per la Ricerca sul Cancro, Genova, Italy; 10 Department of Human Anatomy and Histology, University of Bari, Bari, Italy; 11 Laboratory of Oncology, G. Gaslini Institute, Genova, Italy; The University of Hong Kong, Hong Kong

## Abstract

**Background:**

Non small cell lung cancer (NSCLC) is a leading cause of cancer death. We have shown previously that IL-12rb2 KO mice develop spontaneously lung adenocarcinomas or bronchioalveolar carcinomas.

Aim of the study was to investigate i) IL-12Rβ2 expression in human primary lung adenocarcinomas and in their counterparts, i.e. normal bronchial epithelial cells (NBEC), ii) the direct anti-tumor activity of IL-12 on lung adenocarcinoma cells *in vitro* and *vivo*, and the mechanisms involved, and iii) IL-12 activity on NBEC.

**Methodology/Principal Findings:**

Stage I lung adenocarcinomas showed significantly (P = 0.012) higher frequency of IL-12Rβ2 expressing samples than stage II/III tumors. IL-12 treatment of IL-12R^+^ neoplastic cells isolated from primary adenocarcinoma (n = 6) inhibited angiogenesis *in vitro* through down-regulation of different pro-angiogenic genes (e.g. IL-6, VEGF-C, VEGF-D, and laminin-5), as assessed by chorioallantoic membrane (CAM) assay and PCR array. In order to perform *in vivo* studies, the Calu6 NSCLC cell line was transfected with the IL-12RB2 containing plasmid (Calu6/β2). Similar to that observed in primary tumors, IL-12 treatment of Calu6/β2^+^ cells inhibited angiogenesis *in vitro*. Tumors formed by Calu6/β2 cells in SCID/NOD mice, inoculated subcutaneously or orthotopically, were significantly smaller following IL-12 *vs* PBS treatment due to inhibition of angiogenesis, and of IL-6 and VEGF-C production. Explanted tumors were studied by histology, immuno-histochemistry and PCR array. NBEC cells were isolated and cultured from lung specimens of non neoplastic origin. NBEC expressed IL-12R and released constitutively tumor promoting cytokines (e.g. IL-6 and CCL2). Treatment of NBEC with IL-12 down-regulated production of these cytokines.

**Conclusions:**

This study demonstrates that IL-12 inhibits directly the growth of human lung adenocarcinoma and targets the adjacent NBEC. These novel anti-tumor activities of IL-12 add to the well known immune-modulatory properties of the cytokine and may provide a rational basis for the development of a clinical trial.

## Introduction

IL-12 is a cytokine that exerts potent anti-tumor activity through a combination of immunostimulatory and anti-angiogenic mechanisms [1]–[3]. The latter are related to induction of IFN-γ, which in turn triggers the release of the anti-angiogenic chemokines CXCL9, CXCL10 and CXCL11. In addition, IL-12 down-regulates the production of the pro-angiogenic molecules VEGF and FGF-2 [4]–[7]. The IL-12 receptor (R) is comprised of two subunits, i.e. the ubiquitous IL-12Rβ1 and IL-12Rβ2 that shows a restricted distribution [8].

We [1], [9] have previously shown that the IL-12RB2 gene, encoding the IL-12R chain essential for IL-12 signal transduction, functions as a tumor suppressor in human neoplastic B cells from various chronic lymphoproliferative disorders and acute lymphoblastic leukemia. We [10] have also demonstrated that IL-12rb2 deficient mice develop spontaneously multiorgan lymphoid infiltrates, systemic IL-6 up-regulation and in the second year of life, lung adenocarcinomas and brochioalveolar carcinomas, possibly in relation to IL-6 over-expression [10]. IL-6 promotes lung cancer growth and metastasis [11], [13] and we [10] have demonstrated that IL-12 dampens IL-6 production in mouse splenocytes.

Taken together, the results obtained with IL-12rb2 deficient mice indicated that IL-12 acts as a gatekeeper from the spontaneous development of lung cancer. By inference, IL-12 may represent a novel therapeutic agent against established human lung carcinomas.

Lung cancer is a leading cause of cancer death worldwide [14]. The large majority of cases are non-small-cell lung cancers (NSCLC) [14]. The distribution of NSCLC histologic subtypes has changed over the past 20 years, with decreased incidence of squamous-cell carcinoma and increased frequency of adenocarcinoma, now accounting for 40% of all lung cancer diagnoses [15]. NSCLC prognosis is still grim [16] and novel therapeutic approaches are warranted.

With this background, we have investigated IL-12R expression and function in human primary lung adenocarcinomas and the direct anti-tumor activity of IL-12 on NSCLC cells *in vitro* and *in vivo*, unraveling the molecular mechanisms involved. We have also addressed the question of whether normal bronchial epithelial cells (NBEC), that represent potential counterparts of NSCLC cells, express functional IL-12R.

## Results

### Expression of IL-12Rβ2 in human lung adenocarcinomas

We first investigated the expression of the IL-12Rβ2 in lung tissue samples from seventy lung adenocarcinoma patients.

**Figure 1:**
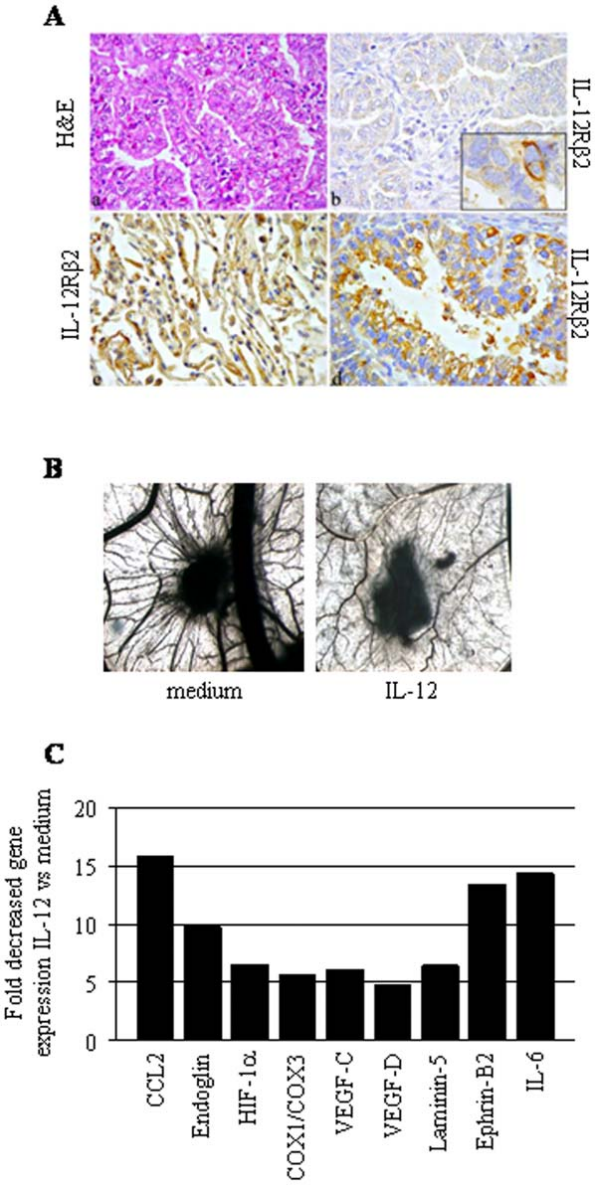
IL-12Rβ2 expression and function in human lung adenocarcinoma. 1A. Histological features and IL-12Rβ2 expression in human bronchioloalveolar lung carcinomas. Non-mucinous bronchioloalveolar carcinoma typically shows columnar neoplastic cells growing along the alveolar septa (a). In 41.4% of adenocarcinomas, neoplastic cells lack IL12Rβ2 expression (b), though a few may sometimes retain it (inset in b). By contrast, alveoli unaffected by malignant process express IL-12Rβ2 (c), as observed in the remaining tumors (d). (×400). 1B. Angiogenic activity of supernatants from one representative lung ADC sample cultured in the presence or absence of hrIL12. CAM treated with sponges loaded with supernatant from the untreated cells were surrounded by allantoic vessels developing radially towards the implant in a ‘spoked-wheel’ pattern (left panel). When supernatants from hrIL-12 treated lung ADC sample was tested, a significant reduction (P = 0.001) of the angiogenic response was appreciable (right panel). These experiments were repeated three times. Original magnification: ×50. 1C. Pooled results from human angiogenesis PCR array performed in three lung ADC samples cultured in the presence or absence of hrIL-12 are shown. Histogram shows fold expression changes of genes in primary samples treated with hrIL-12 vs medium.

Immunohistochemical analyses revealed that 29/70 lung adenocarcinomas (41.4%), 22/49 of which were adenocarcinomas with bronchioloalveolar features (ADBF), 4/13 pure bronchioloalveolar carcinomas (BAC) and 3/8 conventional adenocarcinomas (ADC) [15], did not express IL-12Rβ2 (Table 1 and Fig. 1A, panel b). IL-12Rβ2 was expressed by some of the cells forming the neoplastic glands (inset in panel b). Normal lung peri-tumoral tissue, i.e. alveolar (Fig. 1A, panel c) and bronchiolar (not shown) epithelium, expressed IL-12Rβ2.

**Table 1:** Clinicopathological Characteristics of 70 Patients with Lung Adenocarcinoma and IL-12Rβ2 Expression Profiles of their Tumors

TNM	IL-12Rβ2 Immunostaining *			
	Negative (n29)	Positive (n30)	Weakly Positive (n6)	Mixed (n5)
**Age (range 31–83)**				
**Gender**				
Male (54)	24	22	5	3
Female (16)	5	8	1	2
**Histological type**				
ADBF (49)	22 (45%)	21(43%)	4 (8%)	2 (4%)
BAC (13)	4 (31%)	7 (54%)	1 (7.5%)	1 (7.5%)
ADC (8)	3 (37.5%)	2 (25%)	1 (12.5%)	2 (25%)
**Tumor Size**				
T1 (42)	17	19	4	2
T2 (20)	7	9	2	2
T3 (8)	5	2		1
**Node Status**				
N0 (50)	18	27	2	3
N1 (18)	8	4	4	2
N2 (2)	2			
**Metastases**				
M0 (70)	ND	ND	ND	ND
**Stage**				
IA (33)	12 (36%)	17 (52%)	2 (6%)	2 (6%)
IB (12)	3 (25%)	8 (67%)		1 (8%)
IIA (8)	4 (50%)	2 (25%)	2 (25%)	
IIB (12)	6 (50%)	3 (25%)	2 (17%)	1 (8%)
IIIA (5)	4 (80%)			1 (20%)

* IL-12Rβ2 immunostaining was scored as negative, positive, weakly positive or mixed as described in methods.

ND: not detected.

ADBF: adenocarcinomas with bronchioloalveolar features.

BAC: bronchioloalveolar carcinomas.

ADC: adenocarcinomas.

Thirty/70 adenocarcinomas (42.9%), 21/49 of which were ADBF, 2/8 ADC and 7/13 BAC, were positive for IL-12Rβ2 expression (Table 1 and Fig. 1A, d). 6/70 cases (8.6%), 4/49 of which were ADBF, 1/8 ADC and 1/13 BAC, were weakly positive, while 5 cases (7.1%), 2/49 of which were ADBF, 2/8 ADC and 1/13 BAC, were mixed (Table 1).

When only IL-12Rβ2 positive and negative lung adenocarcinomas according to the score used were analyzed, IL-12Rβ2 positive tumors were significantly more numerous in stage I than stage II/III patients (P = 0.012, Fisher's exact test).

### IL-12 inhibits the pro-angiogenic activity of human primary lung adenocarcinoma cells through down-regulation of multiple pro-angiogenic genes

Lung cancers are known to release several pro-angiogenic factors and angiogenesis inhibition represents a promising therapeutic target [17]. We then asked whether the angiogenic potential of primary ADC cells was affected by IL-12, similarly to that observed in other tumor models [1], [18], [19]. We therefore incubated IL-12Rβ2 expressing neoplastic cells from six lung ADC patients with hrIL-12 or medium alone and tested the angiogenic activity of culture supernatants in the chorio-allantoic membrane (CAM) assay. CAM treated with sponges loaded with VEGF (positive control) or with supernatants from primary ADC cells were surrounded by allantoic vessels developing radially towards the implant in a ‘spoked-wheel’ pattern. In the representative experiment shown in Fig. 1B, left panel, the mean number of vessels formed in the presence of supernatant from ADC cells was 24±3, while that formed in the presence of VEGF was 28±5 (not shown). No vascular reaction was detected around the sponges upon exposure to human recombinant (hr)IL12 diluted in medium at the same final concentration used to treat tumor cells (mean number of vessels = 7±3 in the presence or absence of hrIL-12, not shown). When the supernatants from hrIL-12 treated tumor cells from the same ADC patients were tested in the CAM assay, a significant (P<0.001) reduction of the angiogenic response was appreciable (mean number of vessels = 13±2) (Figure 1B, right panel), as compared to positive control. Similar results with the same statistical significance were obtained when the five remaining primary ADC cell suspensions were tested. Next, we investigated expression of pro-angiogenic and anti-angiogenic genes in primary ADC cells incubated with IL-12 or medium. Neoplastic cells from three different ADC patients were cultured for 48h in the presence or absence of hrIL-12. RNA was extracted from cultured cells, reverse transcribed and tested by PCR Array.

Fig. 1C shows the pooled results from the 3 samples analyzed. IL-12 treatment down-regulated significantly (P<0.001) mRNA of the pro-angiogenic factors CCL2, endoglin, HIF-1α, COX1/COX3, VEGF-C and –D, laminin-5, Ephrin-B2 and IL-6.

Taken together, these findings demonstrated that IL-12R was functional in human primary ADC cells and that IL-12 treatment damped their pro-angiogenic activity.

### IL-12RB2 expression in human NSCLC cell lines

**Figure 2:**
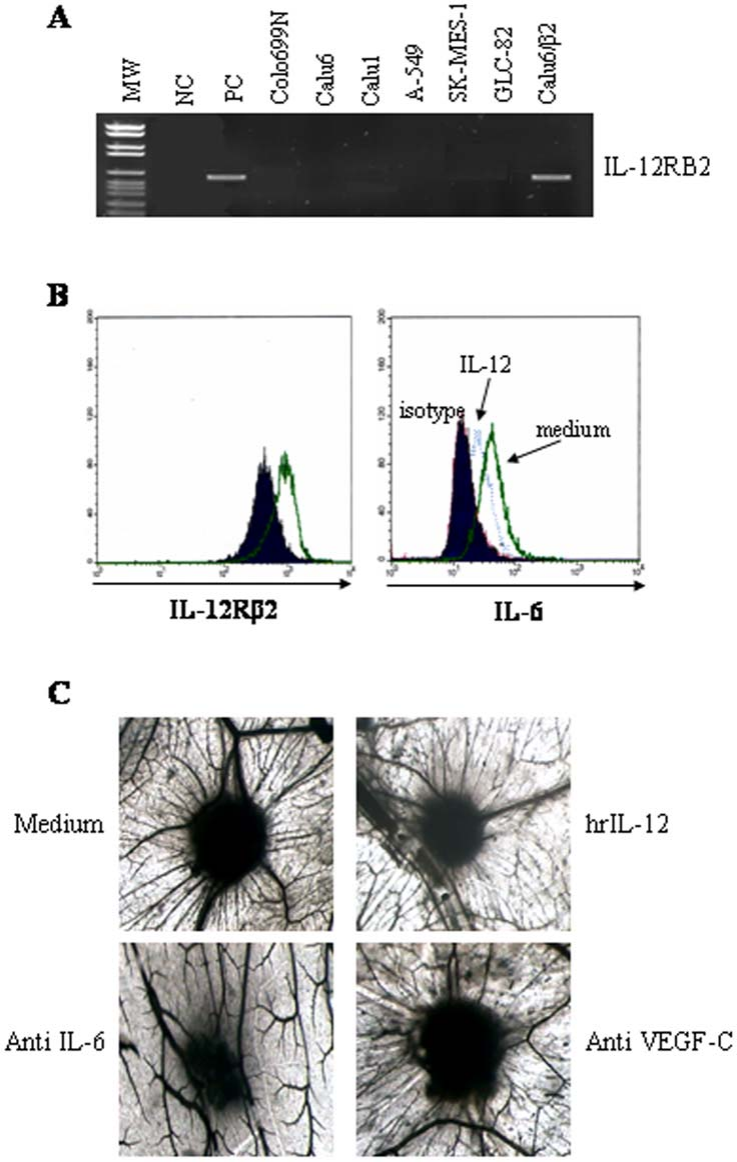
IL-12Rβ2 expression and function in human NSCLC cell lines. 2A. IL-12RB2 expression in NSCLC cell lines, as assessed by RT-PCR. From left to right: MW = molecular weight; NC = negative control (Raji cell line); PC = positive control (total tonsil B cells); different NSCLC cell lines (Colo699, Calu6, Calu1, A549, SK-MES-1, GLC82 and Calu6/β2 cells) are shown. 2B. Left panel. IL-12Rβ2 protein expression in Calu6/β2 cells, as assessed by flow cytometry. Open profile: IL-12Rβ2 staining; dark profile: isotype matched antibody staining. Right panel. IL-6 intracellular staining in Calu6/β2 cells cultured with medium or hrIL-12 for 48 h, as assessed by flow cytometry. Open profile: IL-6 staining in cells cultured with medium; dark profile: isotype matched antibody staining, dashed line: IL-6 staining in cells cultured with IL-12. 2C. Angiogenic activity of supernatants from Calu6/β2 cells cultured with medium alone or hrIL12. CAM treated with sponges loaded with supernatant from the untreated cells were surrounded by allantoic vessels developing radially towards the implant in a ‘spoked-wheel’ pattern (upper left panel). When supernatants from hrIL-12treated Calu6/β2 cells was tested, a significant reduction (P = 0.001) of the angiogenic response was appreciable (upper right panel). Lower panels show the angiogenic activity of Calu6/β2 cells in the presence of an anti-IL-6 mAb (left panel) or of an anti-VEGF-C mAb (right panel). These experiments were repeated three times. Original magnification: ×50.

We next developed a pre-clinical model suitable to investigate the function of IL-12R in human lung adenocarcinoma. To this end, we first screened a panel of human NSCLC cell lines and found that none of them expressed IL-12RB2 mRNA (Fig. 2A) due to methylation of a CpG island in exon 1 (not shown), as previously demonstrated for different malignant cells [1], [9], [20]. Therefore, the Calu6 cell line was stably transfected with an IL-12Rβ2 containing plasmid or with the empty vector containing plasmid (control). From now onwards Calu6 cells transfected with IL-12Rβ2 containing plasmid will be referred to as Calu6/β2. Calu6/β2 cells expressed IL-12RB2 transcript (Fig. 2A) and protein (Fig. 2B, left panel). Incubation of Calu6/β2 cells with hrIL-12 caused i) down-regulation of constitutive IL-6 production (P = 0.002), as assessed by flow cytometry (Fig. 2B, right panel) and ii) significant (P = 0.001) reduction of angiogenic activity in culture supernatants, as assessed by CAM assay (IL-12: mean number of vessels = 15±3, medium: mean number of vessels = 27±4) (Fig. 2C, upper panels).

IL-12 did not modulate production of angiogenic factors or IL-6 by empty vector transfected Calu6 cells (not shown).

Apoptosis and proliferation were not affected by IL-12 treatment (not shown).

These results are consistent with those obtained with human primary ADC cells, thus indicating that the Calu6/β2 cells display functional similarities to the primary ADC cells and represent an useful model for *in vivo* studies.

### IL-6 is a major angiogenic factor involved in vessel formation derived from NSCLC

In order to prove unambiguously that IL-6 and VEGF-C were the major angiogenic factors produced by human NSCLC, we tested the angiogenic activity of Calu6/β2 cell supernatants following incubation with neutralizating antibodies to VEGF-C or IL-6. As shown in Figure 2D, neutralization of IL-6 (left panel) but not of VEGF-C (right panel) inhibited significantly (P<0.001) the angiogenic potential of the Calu6/β2 cells (medium, mean number of vessels = 24±3; anti IL-6, mean number of vessels = 10±3; anti VEGF-C mean number of vessels = 20±4). These results demonstrated unambiguously that IL-6, but not VEGF-C, plays a key role in inducing new vessel formation derived from NSCLC cells. It is of note that VEGF-C is involved in tumor lymphangiogenesis rather than in tumor angiogenesis [21]–[22] and the CAM assay allows to evaluate blood vessel but not lymphatic vessel formation.

### IL-12 inhibits tumorigenicity of Calu6/β2 cells in SCID-NOD mice

**Figure 3:**
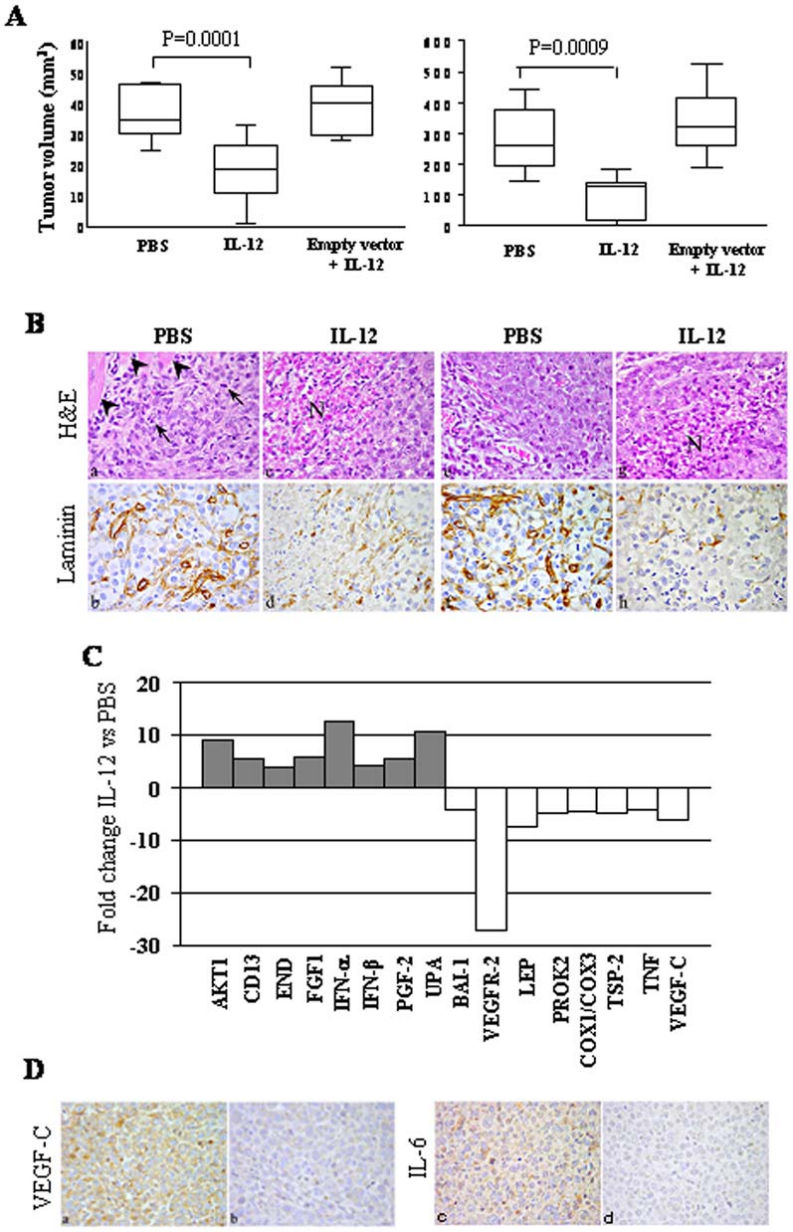
Anti-tumor activity of IL-12 on NSCLC *in vivo.* 3A. Volume of tumors grown after Calu6/β2 cell inoculation orthotopically (left panel) or subcutaneously (right panel) in PBS and hrIL-12 treated animals. Animals injected orthotopically were sacrificed after 23 days, those injected subcutaneously after 14 days. Volume of tumors grown after inoculation orthotopically (left panel) or subcutaneously (right panel) of Calu6 cell transfected with the empty vector hrIL-12 treated animals was also shown (empty vector+IL12). The differences in size between tumors removed from PBS and hrIL-12 treated mice were evaluated by Mann-Whitney U test. Boxes indicate values between the 25^th^ and 75^th^ percentiles, whisker lines represent highest and lowest values for each group. Horizontal lines represent median values. 3B. Tumors (developed after s.c injection) injected subcutaneously with Calu6/β2 cells in PBS-treated SCID/NOD mice are mostly formed of nests of undifferentiated, pleomorphic and proliferating cells (mitotic (figures) features indicated by arrows) rapidly infiltrating the underlying muscle layers (arrowheads) (a), and supplied by a distinct microvessel network, as assessed by laminin staining (b). In hrIL-12 treated mice, tumor histology is altered by the appearance of large areas of ischemic-hemorrhagic necrosis (N) (c) associated with defective microvascular architecture (d) (×400). Orthotopical injection of Calu6/β2 cells gave rise, in PBS-treated mice, to tumors with istopathological features (e) similar to those of subcutaneously developed tumors (a) and supplied by a well developed microvascular network (f). As observed in subcutaneous tumors, in orthotopic tumors as well hrIL-12 treatment induced wide necrosis (g) and severe microvascular alterations (h) (×400). 3C. Human Angiogenesis PCR Array on tumors explanted from hrIL-12 *vs* PBS treated animals 23 days after orthotopic inoculation of Calu6/β2 cells. Histogram shows fold expression changes of genes in tumors from hrIL-12 *vs* PBC treated mice. 3D. Tumors from PBS-treated mice express VEGF-C (a) and IL-6 (c). Expression of VEGF-C and IL-6 is strongly reduced (b and d, respectively) in tumors from hrIL-12 treated mice. (×400).

Tumorigenicity of Calu6/β2 cells or Calu6 cells transfected with empty vector was next investigated. SCID-NOD mice receiving intrapulmonary inoculation of Calu6/β2 cells (orthotopic model) and treated with hrIL-12 developed tumors significantly smaller (P<0.0001) than mice inoculated with the same cells and treated with PBS (n = 7 for both groups; IL-12 treated, median volume 18.55 mm^3^; range 1–32.8 mm^3^. PBS treated, median volume 34.44 mm^3^; range 24.7–46.62 mm^3^) (Figure 3A, left panel).

Mice injected subcutaneously with Calu6/β2 cells and treated with hrIL-12 developed tumors significantly smaller (P = 0.0001) than mice treated with PBS (n = 12 for both groups; IL-12 treated, median volume 131 mm^3^; range 1–186 mm^3^. PBS treated, median volume 261 mm^3^; range 147–3069 mm^3^) (Figure 3A, right panel).

Empty vector transfected Calu6 cells and Calu6/β2 cells produced tumors of similar size in mice treated with PBS (not shown). Furthermore, tumors formed by empty vector transfected Calu6 cells in hrIL-12 treated mice were of similar size to those formed by Calu6/β2 cells in PBS-treated mice independent of the subcutaneous or orthotopic models tested (Fig. 3A, left and right panels).

Tumors formed by Calu6/β2 cells subcutaneously or orthopically in PBS treated mice (Fig. 3B, panels a and e, respectively) were composed of nests of undifferentiated, pleomorphic and proliferating cells invading the underlying muscle layers and supplied by a distinct network of mature microvessels (Fig. 3B, panels b and f, respectively). In contrast, subcutaneous or orthotopic tumors from hrIL-12 treated mice showed large areas of ischemic-hemorrhagic necrosis (Fig. 3B, panel c and g, respectively) associated with defective microvascular architecture (Fig. 3B, panels d and h, respectively).

*In vivo* modulation of the expression of angiogenesis related genes was next investigated by PCR array comparing three tumors from IL-12 treated mice to three tumors from PBS treated mice. IL-12 treatment downregulated significantly (P<0.001) mRNA of the pro-angiogenic factors VEGF-C, VEGFR-2, Leptin, PROK2, COX1/COX3, and thrombospondin 2, whereas up-regulated mRNA of the angiogenesis inhibitors IFN-α and – β (P<0.001). The transcripts of the pro-angiogenic AKT1, CD13, Endoglin, FGF-1, PGF-2 and UPA genes were also up-regulated in these cells (P<0.001) (Fig. 3C).

Finally, tumors from PBS-treated mice expressed VEGF-C (Figure 3D, panel a) and IL-6 (Figure 3D, panel c), as assessed by immunohistochemistry. Expression of VEGF-C and IL-6 was strongly reduced (Figure 3D, panels b and d, respectively) in tumors from hrIL-12 treated mice.

### Expression and function of IL-12Rβ2 in normal human bronchial epithelial cells

Since alveolar and bronchiolar epithelium surrounding the neoplastic tissue from lung adenocarcinoma patients expressed IL-12Rβ2 (Fig. 1A, panel c), we next investigated whether IL-12Rβ2 was expressed and functional in NBEC from patients undergoing lung resection for non-malignant disorders. These cells were expanded in vitro under conditions that allow to preserve functional features similar to those of primary bronchial epithelial cells [23], [24].

**Figure 4:**
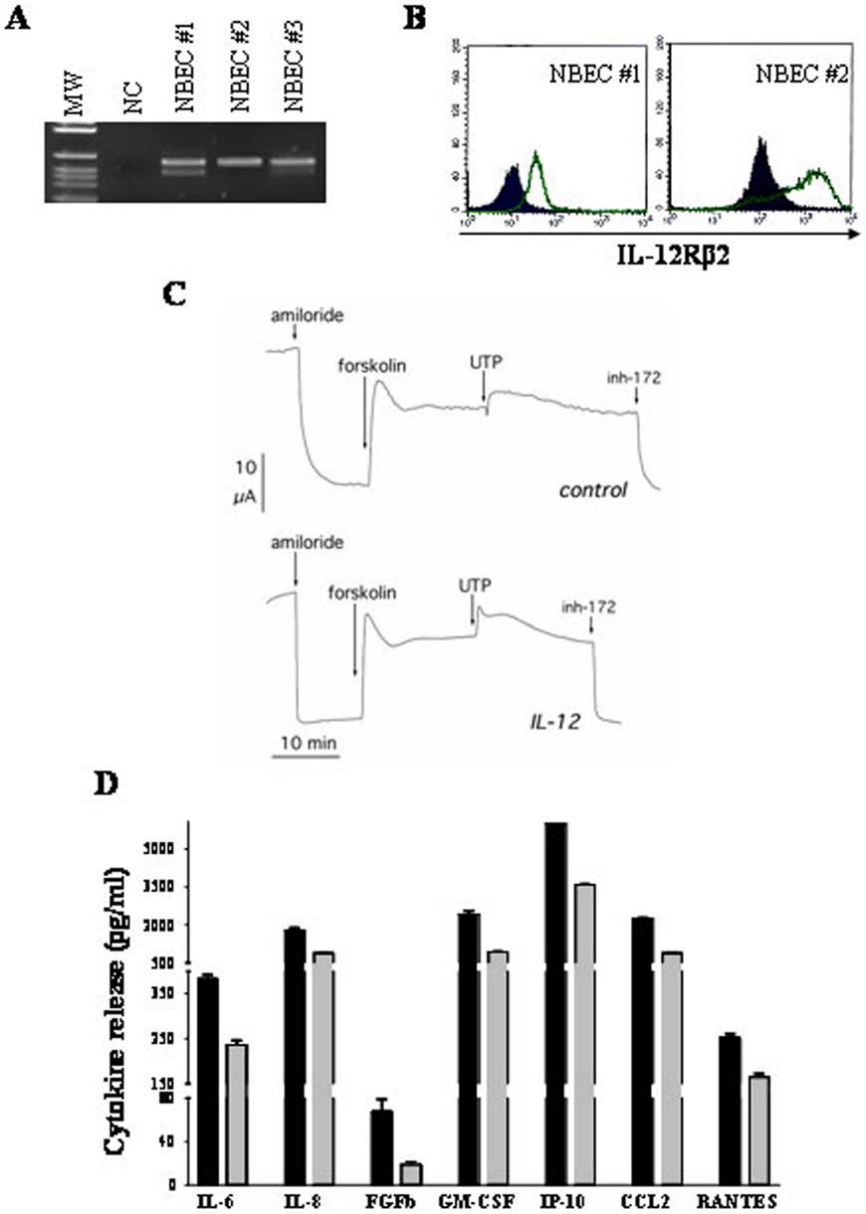
IL-12Rβ2 expression and function in normal bronchial epithelial cells. 4A. IL-12RB2 expression in human primary bronchial epithelial cells, as assessed by RT-PCR. From left to right: MW = molecular weight; NC = negative control (Raji cell line); three different NBEC cultures (NBEC #1, #2 and #3) are shown. 4B. IL-12Rβ2 surface expression in human NBEC, as assessed by flow cytometry. Open profile: IL-12Rβ2 staining; dark profile: isotype matched mAb staining. 4C. Short circuit current recordings in normal bronchial epithelial cells. The figure depicts two representative experiments from control (*top*) and IL-12 treated (*bottom*) epithelia showing responses to amiloride (10 µM, apical), forskolin (20 µM, apical and basolateral), UTP (100 µM, apical), and CFTR_inh_-172 (10 µM, apical). 4D. Cytokine release by human NBEC, as assessed by Bio-Plex Assay. Pooled results from supernatants of three different bronchial epithelial cell suspensions are shown. IL-12 treatment reduced significantly the release of IL-6 (P = 0.0049), IL-8 (P = 0.0071), FGF-b (P = 0.0231), GM-CSF (P = 0.0028), IP-10 (P<0.0001), MCP-1 (P = 0.0002) and RANTES (P = 0.0108).

Figure 4 shows expression of IL-12RB2 mRNA (panel A) and protein (panel B) in cultured bronchial epithelial cells from 3 different donors. These cells also expressed the ubiquitous IL-12RB1 mRNA and protein (not shown).

Our [25], [26] previous studies demonstrated that inflammatory cytokines affected transepithelial ion transport. In particular, we showed that 24–48 h treatment with IFN-γ caused marked changes in Na^+^, Cl^−^, and fluid transport in the bronchial epithelium, possibly by altering the activity/expression of the corresponding ion channels [25]. In order to investigate whether also IL-12 influenced the ion transport properties of NBEC, the latter cells were exposed for 24 h to IL-12 and tested for ion transport.

Cells were sequentially incubated with amiloride to inhibit the epithelial sodium channel (ENaC), the cAMP-elevating agent forskolin to activate cystic fibrosis transmembrane conductance regulator (CFTR), and uridine tri-phosphate (UTP) to trigger the activation of Ca^2+^-dependent Cl^−^ channels [25], [26]. No significant differences in the changes elicited by the three pharmacological agents between control and IL-12 treated cells were detected (Fig. 4C). Indeed, the amiloride-sensitive current, reflecting the activity of ENaC, was 20.5±2.4 µA/cm^2^ (n = 3) for control and 17.8±3.6 µA/cm^2^ (n = 3) for treated cells. Similarly, the cAMP-activated current was not altered: 8.7±4.5 and 7.1±4.1 µA/cm^2^ for control and treated cells, respectively. The lack of effect on cAMP-activated Cl^−^ transport, i.e. on CFTR activity, was also indicated by the similar extent of current change produced by CFTR_inh_-172, a selective inhibitor of the CFTR channel (Fig. 4C). Finally, the effect of UTP, measured at the peak, was 4.2±1.4 µA/cm^2^ (n = 3) for untreated cells and 5.9±0.5 µA/cm^2^ (n = 3) for IL-12 treated cells.

Next, three different NBEC populations were cultured for 48 h with or without IL-12 and supernatants tested for the presence of a panel of cytokines and chemokines. As shown in Figure 4D, IL-12 reduced significantly the constitutive release of the pro-angiogenic factors IL-6 (P = 0.0049), IL-8 (P = 0.0071) and FGF-b (P = 0.0231) and of the pro-inflammatory molecules GM-CSF (P = 0.0028), CCL2/MCP-1 (P = 0.0002) and CCL5/RANTES (P = 0.0108). Finally, IL-12 down-regulated the release of the anti-angiogenic molecule CXCL10/IP-10 (P<0.0001).

## Discussion

In this study, we have addressed the role of the IL-12/IL-12R system in human lung ADC based upon our previous finding that IL-12rb2 KO mice, which produce but cannot utilize IL-12, develop spontaneously systemic IL-6 overproduction and lung ADC or bronchoalveolar carcinoma [10]. Other studies from our group lend cogent support to the notion that IL-12 acts a negative regulator of the growth of both hematopoietic and non-hematopoietic tumors [1], [9], [10], [18], [19].

We first investigated IL-12Rβ2 expression on human primary ADC using immunohistochemistry on a large panel of ADC tissue sections. These studies showed that stage I tumors displayed a significantly higher frequency of IL-12Rβ2^+^ samples than stage II/III tumors, suggesting that IL-12Rβ2 down-regulation may be a tumor escape mechanism. Six primary ADC cell suspensions selected on the ground of high level IL-12Rβ2 expression were next cultured with IL-12 and tested for proliferation, survival and production of angiogenic factors in the CAM assay. These experiments demonstrated unambiguously that the IL-12R expressed by primary ADC cells was functional since their incubation with IL-12 strongly damped angiogenic activity while unaffecting cell proliferation or survival. PCR array experiments with primary ADC cell fractions demonstrated that IL-12 induced predominantly a potent down-regulation of the expression of pro-angiogenic genes (e.g. IL-6, CCL-2 and VEGF). In this respect, supernatants from Calu6/β2 cells incubated with a neutralizing antibody to IL-6 showed a significantly decreased pro-angiogenic activity in the CAM assay, indicating that IL-6 was a major angiogenic factor for NSCLC. In contrast, neutralization of VEGF-C did not affect the angiogenic activity of Calu6/β2 cell supernatants, a finding possibly related to the key role of this molecule in lymphangiogenesis, that cannot be evaluated by the CAM assay.

In order to investigate the *in vivo* effects of IL-12 on human ADC cells, we developed an experimental model based upon the Calu6 cell line transfected with an IL-12Rβ2 cDNA containing plasmid. *In vitro* and *in vivo* studies demonstrated that hrIL-12 decreased significantly IL-6 production and the angiogenic potential of Calu6/β2 cells *in vitro*, while unaffecting proliferation and apoptosis. These results indicated that Calu6/β2 cells represented a model mimicking primary ADC cells and were therefore suitable for *in vivo* experiments.

Therefore, we next investigated the *in vivo* effects of hrIL-12 on the tumorigenicity of Calu6/β2 cells injected orthotopically or subcutaneously in SCID/NOD mice. These animal models allow to assess the direct effects of human IL-12, that is species-specific and inactive in the mouse, on human tumor cells in the absence of immune response [8]. Moreover, the orthotopic model mimicks closely the pattern of growth of human lung adenocarcinoma and provides insights of translational relevance.

These experiments demonstrated that tumors formed by Calu6/β2 cells in SCID/NOD mice, either orthotopically or subcutaneously, were significantly smaller following hrIL-12 *vs* PBS treatment and that the IL-12 mediated anti-tumor activity was primarily due to inhibition of angiogenesis resulting from a complex modulation of the expression of anti-angiogenic (e.g. IFN-α and –β) and pro-angiogenic (e.g. VEGF-C, VEGFR-2, Leptin, PROK2, COX1/COX3, thrombospondin 2) genes. In addition, IL-12 damped *in vivo* expression of VEGF-C and IL-6 proteins [11], [27], [28].

High IL-6 levels have been detected in serum from lung adenocarcinoma patients and correlated with poor prognosis. Furthermore, IL-6 has been implicated in autocrine loops promoting tumor growth. These observations have led to conclude that IL-6 plays an oncogenic role in NSCLC [29].

Our results together with those from other groups indicate that IL-12 can target the tumor microenvironment by acting at different and complementary levels. Thus, we show that IL-12 inhibits directly human NSCLC growth, and others [30] have demonstrated in mouse models that IL-12 alters the functional profile of tumor associated macrophages (TAM) by rapidly reducing production of IL-10, MCP-1/CCL2, migration inhibitory factor and TGF-β1, and increasing release of TNF, IL-15 and IL-18. The ability of IL-12 to reverse TAM function may contribute to amplify subsequent tumor lysis by cytotoxic effector cells [30].

In this connection, we investigated the functionality of IL-12R on *in vitro* expanded NBEC since immunohistochemical studies disclosed IL-12Rβ2 expression in NBEC surrounding the tumor. IL-12 treatment of NBEC reduced significantly their ability to release some cytokines/chemokines that have been shown to be involved in NSCLC angiogenesis, progression and spreading [31]–[36] such as, for example, MCP-1/CCL2 and IL-6 [37]. MCP-1/CCL2 recruits TAM to the tumor site, and these cells release IL-6 and contribute to tumor stroma formation and angiogenesis [37]. On the other hand, IL-12 down-regulated NBEC production of CXCL10, pointing to the complex modulation of angiogenesis-related gene expression operated by the cytokine.

The results here obtained using *in vitro* cultured NBEC conceivably apply also to primary NBEC since cells expanded under our conditions retain functional characteristics similar to those of primary cells. IL-12 mediated down-regulation of the release of tumor promoting cytokines by NBEC may suggest that i) peritumoral NBEC contribute to support lung adenocarcinoma growth [38] and consequently ii) NBEC represent an additional IL-12 target.

This study demonstrates that IL-12R expressed on the surface of tumor cells and of the adjacent NBEC may represent a novel therapeutic target for human lung adenocarcinoma. In principle, IL-12 might be administered to lung adenocarcinoma patients (selected on the ground of IL-12Rβ2 expression in tumor cells) as tumor-targeted formulations to act directly on the tumor microenvironment [39] or systemically to take advantage of the immunomodulatory activity of the cytokine [3]. The feasibility of this potential therapeutic approach appears to be reasonable since IL-12 has been tested as investigational drug in patients with different malignancies [30], [40], [41] and its safety and pharmacokinetics profiles are well known.

## Materials and Methods

### Patients and histological studies

Written informed consent was obtained from all patients and the study was approved by the S.S. Annunziata Hospital's Ethical Committee, Chieti, Italy. This investigation conformed with the principles of Helsinki Declaration.

Lung adenocarcinoma tissues were obtained at diagnosis from seventy consecutive untreated patients with resectable tumors and processed in the Anatomical Pathology Department of the "SS. Annunziata" Hospital. Tumor stage was determined according to the Tumor-Node-Metastasis (TNM) staging system [42]. Tumor histology was classified according to the WHO criteria [15].

Paraffin embedded tissues were processed as reported [19] and stained with the following anti-human antibodies: anti-IL-12Rβ2 (goat polyclonal, Santa Cruz, Santa Cruz, CA, USA), anti-Laminin mAb (Biogenex, San Ramon, CA, USA), anti-VEGF-C mAb (Zymed, San Francisco, CA, USA), or anti- IL6 (rabbit polyclonal, Abcam, Cambridge, UK). IL-12Rβ2 immunostaining was defined as “positive” when ≥70% of the tumor cells stained moderately to strongly; “weakly positive” when ≥70% stained weakly; “mixed” when <70%, but ≥40% were stained; “negative” when <40% were stained. Immunostained sections were examined independently by two pathologists with very good agreement [κ value = 0.85 according to ref.[43]].

Primary adenocarcinoma cells were isolated by mincing of fresh tumor fragments obtained at surgery and containing at least 80% malignant cells, as assessed by morphological analysis. Cell suspensions obtained from four stage I and 2 stage II tumors were selected for further studies following cytofluorimetric demonstration of IL-12Rβ2 expression on higher than 70% of malignant cells. All patients (four males, two females, age range 55–70) were untreated at the time of surgical intervention.

### Flow cytometry and apoptosis assay

Cells were stained with anti-IL-12RB2 goat antiserum (Santa Cruz) or anti-IL-6 mAb (Caltag, Burlingame, CA) using the BD Cytofix/Cytoperm™ fixation/permeabilization kit (BD Biosciences) and analyzed by flow cytometry [18]. Apoptosis was assessed by flow cytometry using an annexin V-FITC apoptosis kit (Immunostep, Salamanca, Spain).

### CAM assay

The CAM assay was performed as described [44], [45]. CAM were loaded with: 1 µl PBS (negative control); 1 µl PBS with 250 ng VEGF (R&D Systems, Abingdon, UK) as positive control; 1 µl of medium from Calu6/β2 cells cultured 48 h with or without hrIL-12; 1 µl of medium from empty vector transfected Calu6 cells cultured 48 h with or without hrIL-12; 1 µl of medium from primary ADC cells cultured 48 h with or without hrIL-12, 1 µl of medium containing hrIL-12. All supernatants were tested in quadruplicate and means±SD were calculated. On day 12, the angiogenic response was evaluated by image analyzer as number of vessels converging toward the sponges [44], [45].

In some experiments, CAM were treated on day 8 with sponges loaded with 1 µl of medium from Calu6/β2 cells alone or containing 50 ng of an anti-human IL-6 mAb (Santa Cruz Biotechnology, Santa Cruz, USA) or of an anti-human VEGF-C mAb (Santa Cruz Biotechnology).

### PCR-Array

RNA was extracted using TRIZOL® from Invitrogen (Carlsbad, CA, USA) and reverse transcribed by the ReactionReady™ First Strand cDNA Synthesis kit (SuperArray Bioscience Corporation). Contaminant genomic DNA was removed by Dnase treatment using Rneasy Micro Kit (Qiagen GmbH) before starting PCR-Array procedure.

Human Angiogenesis RT^2^ Profiler™ PCR Array and RT^2^ Real-Time™ SyBR Green/ROX PCR Mix were purchased from SuperArray Bioscience Corporation. PCR was performed as described [18], [19]

### Cell culture and transfection

Human NSCLC A549, Calu-1, Calu-6, Colo699, GLC82, SK-MES-1 and SK-LU-1 cell lines or primary ADC cells were cultured in RPMI 1640 medium (Seromed-BiochromKG, Berlin, Germany) with 10% FCS (Seromed) for the indicated times. Human recombinant hrIL-12 (20 ng/ml) was from Wyeth, Cambridge, MA. Based upon a preliminary screening of cell line tumorigenicity in the orthotopic model (see below), Calu6 cells were selected to be transfected with IL-12RB2/pEGFP-N1 or empty vector by electroporation. Stable transfectants were selected and checked for IL-12RB2 gene and protein expression [1].

### Methylation assay

DNA was extracted using GenElute DNA miniprep kit from Sigma and the methylation status of the target sequence was assessed by Methylation Specific PCR [1].

### Mice studies

Five- to 7-week-old female SCID-NOD mice (Harlan Laboratories, Udine, Italy) were housed in pathogen-free conditions. All procedures were performed according to the National and International current regulations (D.l.vo 27/01/1992, n.116, European Economic Community Council Directive 86/609, OJL 358, Dec. 1, 1987).

Two groups of seven animals each were injected subcutaneously with 3×10^6^ empty vector transfected Calu6 cells. Two additional groups of seven mice each were injected subcutaneously with 3×10^6^ Calu6/β2 cells. One group of mice for each of the above combinations was treated with 3 weekly doses of hrIL-12 subcutaneously (1 µg/mouse/dose) starting from 6 h after injection of tumor cells. The other group of mice from each combination was injected with PBS according to the same time schedule. Fourteen days after tumor cell inoculation, mice were sacrificed and tumor volume measured as described [1].

In another series of experiments, two groups of twelve mice each were anesthetized with a mixture of Ketamine (80 mg/kg body weight) and Xylazine (5 mg/kg body weight) IP. The skin overlying the left chest wall in the mid-axillary line was prepped with tricotomy, disinfection. Incision was performed (3 mm) and the underlying chest wall and intercostal spaces were visualized. 1.5×10^5^ Calu6/2 cells were injected into the left lateral thorax, at the lateral dorsal axillary line. After tumor injection, the mice were turned to the left lateral decubitus position and observed for 45 to 60 min until fully recovered. No anesthesia or surgery-related deaths occurred. Two additional groups of twelve mice were injected orthotopically with 1.5×10^5^ empty vector transfected Calu6 cells. One group of mice from each of the above combinations was treated with 3 weekly doses of hrIL-12 intravenously (1 µg/mouse/dose) or PBS starting from 6 h after injection of tumor cells. Animals were sacrificed twenty three days after tumor inoculation.

### Primary culture of bronchial epithelial cells

Human bronchial epithelial cells were obtained from lung resections performed for non-neoplastic conditions and cultured as previously described [23], [24]. The collection and processing of human cells was approved by the Gaslini Institute's ethics committee. Briefly, bronchial epithelial cells were detached by overnight digestion with protease XIV (Sigma, St. Louis, MO, USA) and cultured on flasks in a serum-free culture medium consisting of a 1∶1 mixture of LHC9 (Invitrogen s.r.l., Milan, Italy), and RPMI 1640 (Euroclone, Paington, Devon, UK). To obtain polarized epithelia, cells were plated at high density (2.5×10^6^ cells/cm^2^) on Transwell porous supports (Corning Costar, Cambridge, MA, USA). After 24 h, the serum free medium was replaced with an enriched mixture containing DMEM/Ham's F12 (1∶1) plus 2% FCS (Invitrogen) and various hormones and supplements [23], [24]. This medium allows the generation of differentiated epithelia with high electrical resistance, resembling the airway epithelium *in vivo*. Cell differentiation was monitored by measuring daily transepithelial electrical resistance and potential difference with an epithelial voltmeter (World Precision Instruments, Sarasota, FL, USA). Twenty ng/ml of hrIL-12 or medium alone were added to apical and basolateral medium 8 days after plating, when the electrical resistance was 800–1000 xcm^2^ and the potential difference was between −40 and −50 mV. Cells were subsequently cultured 24 to 48 h.

### Short-circuit current measurements

Snapwell inserts carrying differentiated monolayers of bronchial epithelial cells were mounted in a self-contained Ussing chamber system (Vertical diffusion chamber; Corning Life Sciences) 8 days after cell plating. Where needed, IL-12 (20 ng/ml) was applied in the culture medium (apical and basolateral) for 24 hours before Ussing chamber experiments were performed. The apical and basolateral chambers contained identical solutions (in mM): 126 NaCl, 0.38 KH_2_PO_4_, 2.1 K_2_HPO_4_, 1 MgSO_4_, 1 CaCl_2_, 24 NaHCO_3_ and 10 glucose (Sigma). During experiments, solutions in both chambers were continuously bubbled with a mixture of 5% CO_2_/95% air and all measurements were done at 37°C. Cells were sequentially incubated with amiloride (10 µM, apical solution) to inhibit the epithelial sodium channel (ENaC), the cAMP-elevating agent forskolin (20 µM, apical and basolateral solution) to activate CFTR, and UTP (100 µM, apical solution) to trigger the activation of Ca^2+^-dependent Cl^−^ channels [25], [26]. All of these reagents were from Sigma. The apical and basolateral chambers were connected to a DVC-1000 voltage clamp amplifier (World Precision Instruments, Inc., Sarasota, FL) using Ag/AgCl electrodes and 1 M KCl agar bridges. Short circuit currents, reflecting transepithelial ion transport, were digitized using a PowerLab 4/25 data acquisition system.

### ELISA

Culture supernatants were tested in triplicate using the human twenty seven cytokine Bio-Plex Assay (Bio-Rad Laboratories, Inc., CA, USA).

### Statistical methods

Analysis of correlations between tumor stage and IL-12Rβ2 immunostaining was carried out using Fisher's exact test with 99% confidence interval. Differences vessel numbers or cytokine concentrations were evaluated by Student's *t* test. Differences in tumor volume were calculated using Mann-Whitney test comparing two independent samples, with 99% confidence interval.

All statistical tests were two tailed. A P value lower than 0.05 was considered statistically significant.
